# Assessment of the Genetic Diversity of Different Job's Tears (*Coix lacryma-jobi* L.) Accessions and the Active Composition and Anticancer Effect of Its Seed Oil

**DOI:** 10.1371/journal.pone.0153269

**Published:** 2016-04-12

**Authors:** Xiu-Jie Xi, Yun-Guo Zhu, Ying-Peng Tong, Xiao-Ling Yang, Nan-Nan Tang, Shu-Min Ma, Shan Li, Zhou Cheng

**Affiliations:** 1 School of Life Science and Technology, Tongji University, Shanghai, China; 2 College of Pharmacy, Zhejiang University of Technology, Hangzhou, Zhejiang, China; Technical University in Zvolen, SLOVAKIA

## Abstract

Job’s tears (*Coix lachryma-jobi* L.) is an important crop used as food and herbal medicine in Asian countries. A drug made of Job’s tears seed oil has been clinically applied to treat multiple cancers. In this study, the genetic diversity of Job’s tears accessions and the fatty acid composition, triglyceride composition, and anti-proliferative effect of Job’s tears seed oil were analyzed using morphological characteristics and ISSR markers, GC-MS, HPLC-ELSD, and the MTT method. ISSR analysis demonstrated low genetic diversity of Job’s tears at the species level (*h* = 0.21, *I* = 0.33) and the accession level (*h* = 0.07, *I* = 0.10), and strong genetic differentiation (G_ST_ = 0.6702) among all accessions. It also clustered the 11 accessions into three cultivated clades corresponding with geographical locations and two evidently divergent wild clades. The grouping patterns based on morphological characteristics and chemical profiles were in accordance with those clustered by ISSR analysis. Significant differences in morphological characteristics, fatty acid composition, triglyceride composition, and inhibition rates of seed oil were detected among different accessions, which showed a highly significant positive correlation with genetic variation. These results suggest that the seed morphological characteristics, fatty acid composition, and triglyceride composition may be mainly attributed to genetic factors. The proportion of palmitic acid and linoleic acid to oleic acid displayed a highly significant positive correlation with the inhibition rates of Job’s tears seed oil for T24 cells, and thus can be an important indicator for quality control for Job’s tears.

## Introduction

Job’s tears (*Coix lachryma-jobi* L.), which belongs to the *Coix* genus, the Andropogoneae tribe, and the Gramineae family [1], is an important crop used as food and herbal medicine in Asian countries. The Job’s tears seed is traditionally used as a diuretic, digestive tonic, analgesic, antispasmodic and anti-inflammatory agent in China [2]. Job’s tears is native to and extensively cultivated in south Asia [3], and now it has been introduced to almost all tropical and subtropical zones of the world [4]. In China, the wild Job’s tears resource is decreasing rapidly along with the economic advance [5].

Job’s tears seeds have been reported to have anti-cancer [6–10], hypolipidemic [11,12], hypoglycemic [11,12], antioxidant [13–15], anti-inflammatory [15–17], and anti-allergic [18,19] properties. An anticancer drug made of Job’s tears seed oil, that is, Kanglaite, has been applied clinically in China and proven effective in treating multiple cancers [20–23]. Kanglaite is the first drug derived from a traditional Chinese herbal remedy that was approved by the USA Food and Drug Administration to undergo clinical trials in the United States [24]. The demand for Job’s tears is increasing rapidly with its medical use, but knowledge on genetic diversity and quality evaluation of Job’s tears is still limited.

Previous studies of Job’s tears have focused mainly on its agronomic characteristics [25–27], chemical components [15–17], and pharmacological functions [6,11–15]. Studies on genetic variation and relationships of Job’s tears have been made on accessions mainly from the Guangxi, Guizhou, and Yunnan Provinces in southern China [28–30]. The main active components of Job’s tears seed oil were triglycerides containing palmitic, stearic, oleic, and linoleic acid [31]. Some methods such as pre-column derivation HPLC [32], GC-MS [33], HPLC-MS [34], and HPLC-ELSD [35] were developed to qualify and quantify the fatty acid and triglyceride components of Job’s tears. However, the differences in chemical composition and antitumor efficacy among Job’s tears accessions from different regions are still not clear. Moreover, no effort has been made to assess the relationship between genetic diversity of accessions and chemical composition and anti-proliferative effects of the seed oil for different Job’s tears accessions. These prevent the proper utilization of Job’s tears.

Molecular markers such as RAPD (random amplified polymorphic DNA), AFLP (amplification fragment length polymorphism) and ISSR (inter-simple sequence repeat) are useful tools to evaluate the genetic variation of plants. Among these markers, ISSR is attractive because it requires no previous DNA sequence information and is highly variable, reproducible, and cost effective [36,37]. ISSR has been widely used to study the genetic diversity of various plants at low taxonomic levels (species, populations or even clonal) [38–41]. So ISSR markers were selected to analyze the genetic diversity of Job’s tears.

The objectives of this study were to (i) analyze the genetic diversity of 11 Job’s tears accessions from the main distribution regions in China based on morphological characteristics and ISSR markers; (ii) detect the fatty acid and triglyceride compositions of seed oil from different accessions using GC-MS and HPLC-ELSD; (iii) assess the anti-proliferative effect of seed oils from different accessions on T24 cells using the MTT method; and (iv) clarify the relationship between genetic variation and active component composition of Job’s tears seeds, and further impact on anti-proliferative effect. Understanding the genetic diversity and indicators of quality could provide valuable information for the utilization of, breeding of, and conservation strategy establishment for Job’s tears.

## Materials and Methods

### Plant materials

**Fig 1:**
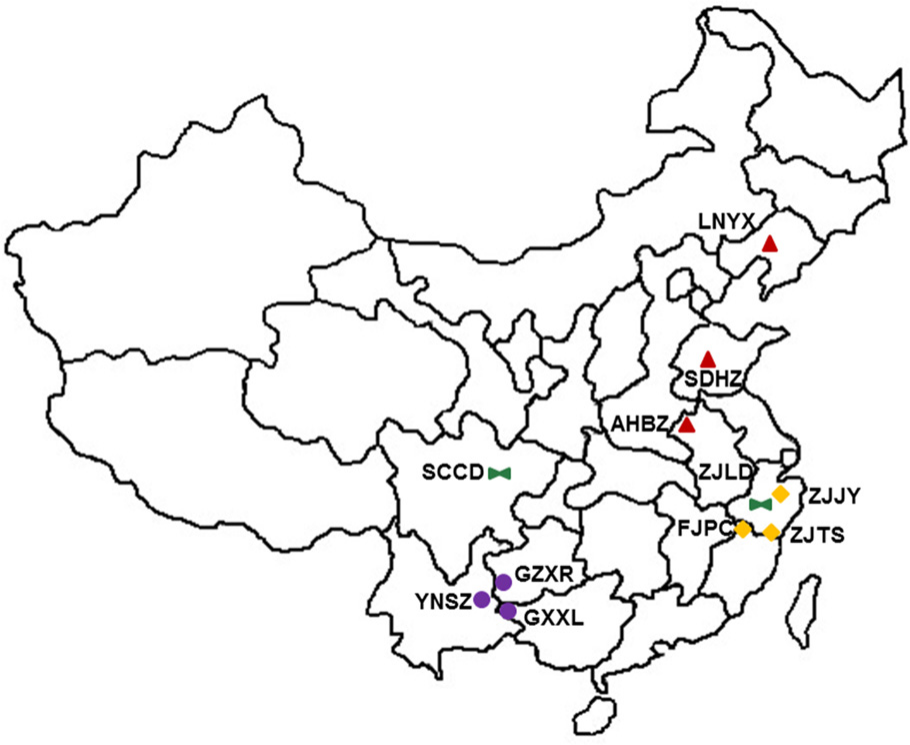
Geographic localities of Job’s tears accessions used in this study. Accession codes were defined in Table 1.

Eleven Job’s tears accessions were included in this study (Fig 1, Table 1). Nine cultivated accessions were sampled from eight provinces of the three main distribution regions in China (i.e., northern China, central China, and southern China). Two wild accessions were collected from Liandu (Zhejiang province) and Chengdu (Sichuan province). In total, 50 plants per accession and 20–30 seeds per plant were sampled. Fifteen seeds with 1 seed per plant were randomly selected for seed morphological analysis, 20 seeds with 1 seed per plant were randomly selected for ISSR analysis, and the rest seeds were dehulled and ground into a powder together for HPLC-ELSD analysis, GC-MS analysis and MTT assay. No special permit was required for the sampling in this study. All samples were collected by researchers with introduction letters of IBAT (Institute of Bioresources and Applied Technology), Tongji University. Collected specimens were preserved in the Herbarium of IBAT (Institute of Bioresources and Applied Technology), Tongji University, Shanghai, China.

**Table 1:** Codes and geographic localities of Job’s tears accessions used in this study.

Accession Codes	Geographic localities	Longitude (N)	Latitude (E)
SCCD*	Chengdu, Sichuan Province	104°03′44.18″	30°34′21.43″
ZJLD*	Liandu, Zhejiang Province	119°54′30.32″	28°26′57.77″
LNYX	Yixian, Liaoning Province	121°14′01.00″	41°31′51.74″
SDHZ	Heze, Shandong Province	115°28′30.98″	35°14′02.71″
AHBZ	Bozhou, Anhui Province	115°47′38.78″	33°50′22.68″
ZJJY	Jinyun, Zhejiang Province	120°05′12.84″	28°39′43.80″
FJPC	Pucheng, Fujian Province	118°32′11.88″	27°55′13.87″
ZJTS	Taishun, Zhejiang Province	119°42′46.15″	27°33′34.75″
GZXR	Xingren, Guizhou Province	105°10′22.80″	25°26′05.17″
YNSZ	Shizong, Yunnan Province	103°59′16.29″	24°49′53.32″
GXXL	Xilin, Guangxi Province	105°05′26.73″	24°29′32.43″

Abbreviations listed here are those used in all subsequent figures.

*Asterisks indicate wild accessions.

### Morphological analysis

The shape, size, color and texture of seed are important in the classification of *Coix* species and varieties [42,43]. Four qualitative morphological characteristics were measured on 15 seeds (1 seed per plant) of each accession i.e., seed color, seed hardness, surface stripe of seed and color of seed kernel.

Eleven quantitative morphological characteristics were measured on the same 15 seeds of each accession. Seed weight, weight of seed kernel and weight of the hull and the testa were measured with a scale sensitive to 0.0001g. Seed thickness, seed length, seed width, thickness of seed kernel, length of seed kernel and width of seed kernel were measured by a digimatic calipers (0–150 mm, Shanghai Measuring & Cutting Tool Works Co., Ltd., China). The length-width ratio of seed and the corresponding seed kernel were calculated in Excel.

### DNA extraction and ISSR-PCR amplification

The Job’s tears seeds germinated in a light-proof environment until the seedlings reached a height of 3–4 cm. Total genomic DNA was extracted from the seedlings using a modified CTAB (cetyltrimethyl ammonium bromide) method [44], and then dissolved in 1× TE buffer (10 mM Tris-HCl and 1 mM EDTA, pH 8.0) and stored at -20°C. A total of 218 samples, including 20 samples of each accession (SDHZ with 18 samples), were used for the ISSR analysis.

One hundred ISSR primers screened in this study were synthesized by Shanghai Sangon Biological Engineering Technology & Service Co., Ltd. (China) according to the primer set published by the University of British Columbia (UBC, Canada). PCRs were performed in 20 μL reaction mixtures containing 10 mM Tris-HCl (pH 8.8), 50 mM KCl, 2 mM MgCl_2_, 0.2 mM each of dNTPs, 0.2 μM of primers, 0.5 U of Taq DNA polymerase (TaKaRa), and approximately 20 ng of template DNA. The amplifications were performed in a Eppendorf Mastercycler Gradient PCR machine (Eppendorf, Germany) with the following program: an initial denaturation at 94°C for 4 min; 40 cycles of denaturation at 94°C for 45 s, annealing at 50–55°C (depending on different primers) for 45 s, elongation at 72°C for 1.5 min; and a final extension at 72°C for 10 min.

The amplification products were electrophoresed in a horizontal gel apparatus (Bio-Rad, USA) using 1.5% agarose gel in 1× TAE buffer (pH 8.0) at 100 V for 45 min. The gels were stained with 0.8 μg/mL ethidium bromide for approximately 30 min, and then photographed under UV light using the UVP-GDS8000 Gel Documentation System (UVP, USA).

### HPLC-ELSD analysis

Acetonitrile and methylene dichloride were chromatographically pure and purchased from Tedia Company, Inc. (Ohio, OH, USA).

The Job’s tears seeds were dehulled and ground into a powder. One gram of powder was accurately weighed and soaked in 25 mL of mobile phase (a 67:33 ratio of acetonitrile:methylene dichloride, V/V) for 2 h at 25°C; then, it was ultrasonically extracted for 30 min. The extract was diluted to 25 mL and filtered through a Millipore filter (0.45 μm). The filtrate was used for HPLC-ELSD (High Performance Liquid Chromatography- Evaporative Light Scattering Detector) analysis.

HPLC-ELSD analyses were carried out on an Agilent 1200 HPLC system (Agilent Technologies, CA, USA) equipped with an evaporative light scattering detector (ELSD) 3300 (Alltech Associates, IL, USA). An Xtimate^TM^ C18 column (4.6 mm × 250 mm, 5 μm) was maintained at 30°C for analysis. Acetonitrile and methylene dichloride (67:33) were used as the mobile phase with a flow rate of 0.6 mL/min. A drift tube temperature of 45°C and a nebulizer gas (N_2_) flow rate of 1.5 L/min were set as the detection conditions for ELSD. An injection volume of 10 μL was used for quantitative analysis.

### GC-MS analysis

Acetone, sulfuric acid, methanol, ether, sodium sulfate, and n-hexane were analytically pure and purchased from Hangzhou Gaojing Fine Chemical Co., Ltd (Hangzhou, China).

Five grams of dehulled Job’s tears seed powder was extracted by reflux with 50 mL acetone for 1 h. This was done twice. The extracts were combined and evaporated by rotary vacuum at 60°C. Then, the extract was added to a 10 mL solvent of sulfuric acid and methanol (1:9), extracted by reflux at 60°C for 2 h, extracted with ether, washed to a neutral pH with distilled water, dehydrated with sodium sulfate, evaporated to remove the ether, and dissolved with n-hexane to a volume of 5 mL.

GC-MS analyses were carried out on the Agilent 7890A-5975C (Agilent Technologies, CA, USA) with electron impact ionization (70 eV). A DB-5 fused silica capillary column (30 m × 250 μm × 0.25 μm, J&W Scientific, USA) was used. The column temperature was maintained at 60°C for 1 min; then, it was programmed to increase from 60°C to 200°C at a rate of 15°C/min, maintained at 200°C for 5 min, raised to 280°C at a rate of 8°C/min, and finally held at 280°C for 1 min. The carrier gas used was helium, with a flow rate of 1 mL/min and a split ratio of 50:1. Mass range was 50–550 m/z and the injector temperatures were set at 220°C. The components were identified by comparison of mass spectra with those stored in the available database (NIST version 2008).

### Cell culture and MTT assay

Ethanol, sorbitane monooleate (Span 80), and polyoxyethylene-80-sorbitan monooleate (Tween 80) were analytically pure and purchased from Sinopharm Chemical Regent Co., Ltd. (Shanghai, China). MTT and dimethyl sulfoxide (DMSO) were purchased from Shanghai Sangon Biological Engineering Technology & Service Co., Ltd. (China). RPMI-1640 medium (2.05 mM/L-glutamine, without calcium nitrate), new-born calf serum, penicillin, and streptomycin were purchased from Hyclone (Logan, UT, USA). The human bladder carcinoma T24 cells were purchased from ATCC (Manassas, VA, USA).

To obtain the seed oil of Job’s tears, 30 g dehulled seed powder was ultrasonically extracted with 300 mL of 100% ethanol for 1 h, filtered to remove the residue, and evaporated by rotary vacuum. Then, 100 μL oil, 10 μL emulsifier of Span 80 and Tween 80 (2:3), and 890 μL cell medium were mixed and vortexed to obtain a 10% ethanol extract emulsion.

The human bladder carcinoma T24 cells were cultured in RPMI-1640 cell medium supplemented with 10% new-born calf serum, 100 U/mL penicillin, and 100 μg/mL streptomycin at 37°C and a 5% CO_2_ atmosphere.

The MTT assay procedure used was as follows: T24 cells (5 × 10^3^ cells/well) were inoculated into a 96-well polystyrene plate and incubated for 18 h. The test group, emulsifier group, positive group, and control group were treated with 0.5% or 0.33% ethanol extract emulsion, 0.5‰ or 0.33‰ emulsifier, 100 μg/mL MMC (mitomycin C), and pure medium, respectively. After incubation for 24 h, 20 μL MTT (5 mg/mL) was added to each well and incubated for 4 h before removing the medium, and 150 μL DMSO was then added to each well to dissolve the precipitate. The absorbance was measured by a Multifunctional Microplate Reader SpectraMax M5 (Molecular Devices, USA) at a wavelength of 490 nm, and the inhibition rate was calculated according to the following formula:

$$\text{Inhibition Rate}=\frac{\text{OD}490(\text{control})-\text{OD}490(\text{test})}{\text{OD}490(\text{control})}\times 100\%$$

### Data analysis

One-way ANOVA of 11 quantitative morphological characteristics and the principal component analysis (PCA) of all 15 morphological characteristics were performed with SPSS 19.0 (SPSS Inc., USA). The Euclidean distances between Job’s tears accessions based on morphological characteristics were calculated using the between-groups linkage method in SPSS 19.0.

The discernible and reproducible DNA bands ranging from 250 to 2000 bp were scored 1 for presence and 0 for absence to construct the binary data matrix for statistical analysis. The parameters of genetic diversity as percentage of polymorphic fragments (PPF), Neiʼs expected heterozygosity (*h*), Shannon's Information index (*I*), Neiʼs coefficient of accession differentiation (G_ST_), and gene flow among all accessions (N_m_) were calculated using PopGene 1.32 [45]. The unweighted pair-group method with arithmetic means (UPGMA) dendrogram was also generated by PopGene 1.32. The genetic variation within/among accessions was determined by analysis of molecular variance (AMOVA) [46] using GenAlEx 6.2 [47]. Nei’s genetic distances and geographic distances were calculated and used to perform the Mantel test [48]. The correlation between these two distance matrices was investigated using GenAlEx 6.2.

The peak area percentages of triglyceride and fatty acid content were calculated with the area normalization method and used as variables for statistical analysis. One-way ANOVA and PCA of triglyceride and fatty acid content were also performed with SPSS 19.0. The Euclidean distances between accessions, based on triglyceride and fatty acid content, were calculated using the between-groups linkage method in SPSS 19.0.

The one-way ANOVA of inhibition rates was conducted with SPSS 19.0. The correlations between the morphological, genetic, and chemical matrices were examined using the Mantel test of GenAlEx 6.2. SPSS 19.0 was applied in testing the associations of inhibition rate with triglyceride and fatty acid content.

## Results

### Morphological diversity

**Fig 2:**
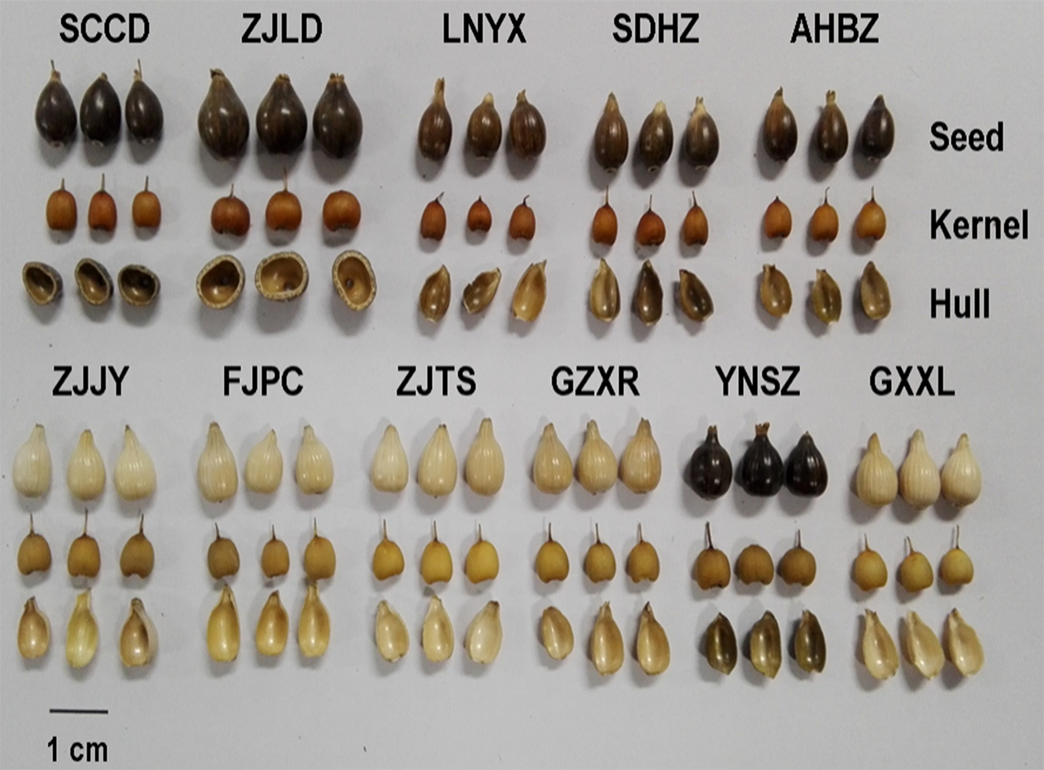
Seed morphological characteristics of Job’s tears accessions. Seed color: brown (SCCD, ZJLD, LNYX, SDHZ, AHBZ and YNSZ), yellow-white (ZJJY, FJPC, ZJTS, GZXR and GXXL); Seed hardness: high (SCCD and ZJLD), moderate (LNYX, SDHZ, AHBZ and YNSZ), low (ZJJY, FJPC, ZJTS, GZXR and GXXL); Surface stripe of seed: absent (SCCD and ZJLD), present (LNYX, SDHZ, AHBZ, ZJJY, FJPC, ZJTS, GZXR, YNSZ and GXXL); Color of seed kernel: red-brown (SCCD, ZJLD, LNYX, SDHZ and AHBZ), yellow (ZJJY, FJPC, ZJTS, GZXR, YNSZ and GXXL); Seed size: big (SCCD and ZJLD), moderate (ZJJY, FJPC, ZJTS, GZXR, YNSZ and GXXL), small (LNYX, SDHZ and AHBZ); Seed shape: round (SCCD and ZJLD), moderate (ZJJY, FJPC, ZJTS, GZXR, YNSZ and GXXL), narrow (LNYX, SDHZ and AHBZ).

The seed qualitative and quantitative morphological characteristics of Job’s tears accessions were presented in S1 Table. Variations were recorded for the 4 qualitative morphological characteristics measured. The seeds of 6 accessions (SCCD, ZJLD, LNYX, SDHZ, AHBZ and YNSZ) are brown, and those of the other 5 accessions are yellow-white (Fig 2). The seed hardness ranged from low to high. Two wild accessions (SCCD and ZJLD) are hard, 4 accessions (LNYX, SDHZ, AHBZ and YNSZ) are medium-hard, and the other 5 accessions (ZJJY, FJPC, ZJTS, GZXR and GXXL) are soft. The 2 wild accessions are polished without a surface stripe, and the 9 cultivated accessions possess surface stripes. The seed kernels of 5 accessions are red-brown, and those of the other 6 accessions are yellow (Fig 2).

One-way ANOVA showed significant differences between accessions for all quantitative seed characteristics. Among the accessions, ZJLD had the highest seed weight (0.283 g), seed thickness (0.687 cm), seed length (1.023 cm), seed width (0.809 cm), weight of seed kernel (0.091 g) and width of seed kernel (0.596 cm), and the minimum values of these 6 characteristics were recorded in AHBZ (0.078 g, 0.453 cm, 0.788 cm, 0.518 cm, 0.050 g and 0.429 cm, respectively) (S1 Table). AHBZ recorded the maximum length-width ratio of seed and seed kernel (1.523 and 1.157, respectively). The minimum length-width ratio of seed and seed kernel were found in YNSZ (1.246) and ZJLD (0.880). GXXL had the highest thickness of seed kernel (0.416 cm) and length of seed kernel (0.544 cm), while the minimum were recorded in AHBZ (0.332 cm) and ZJLD (0.456 cm), respectively. The weight of the hull and the testa ranged from 0.018 g (GZXR) to 0.192 g (ZJLD).

**Fig 3:**
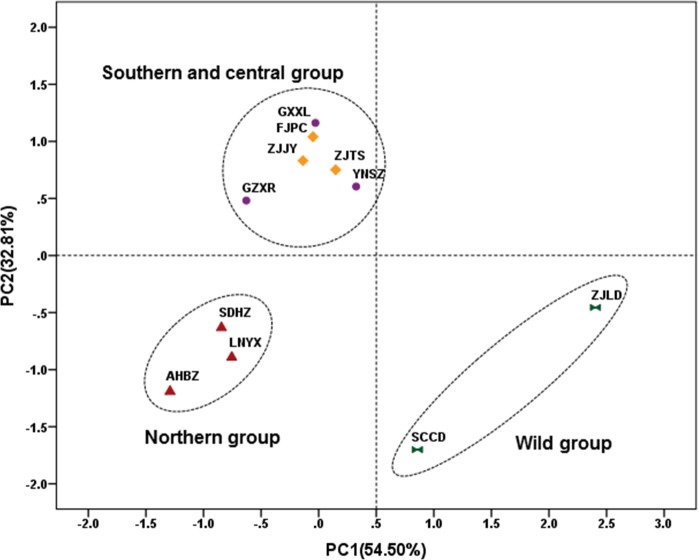
PCA scatter plot of Job’s tears accessions constructed by seed morphological characteristics. Accession codes were defined in Table 1.

In the PCA scatter plot constructed by the 15 morphological characteristics, the Job’s tears accessions can be divided into 3 groups, which corresponded to the southern and central, northern, and wild groups, respectively (Fig 3). The southern and central group contained six cultivated accessions of ZJJY, FJPC, ZJTS, GZXR, YNSZ, and GXXL from southern and central China, which were almost always characterized by yellow-white and soft seeds, surface stripes, and yellow seed kernels. An exception was the YNSZ accession, which possessed brown and medium-hard seeds similar to the accessions of the northern group. Three cultivated accessions of LNYX, SDHZ, and AHBZ from northern China comprised the northern group. These were characterized by brown, medium-hard seeds with surface stripes and red-brown seed kernels. The wild group consisted of two wild accessions of SCCD and ZJLD, which were characterized by brown, hard seeds with red-brown seed kernels and without a surface stripe.

Among the three divided groups, the accessions in the wild group possessed the biggest, heaviest, and roundest seeds. The accessions in the northern group possessed the smallest, lightest, and narrowest seeds. The southern and central group contained accessions with seeds of moderate weight and size. The weight and size of seed kernels were not significantly different among the three groups, whereas the shape of the seed kernel varied from oblate in the wild group to oblong in the northern group (Table 2, Fig 2).

**Table 2:** Mean values of quantitative seed morphological characteristics among the divided Job’s tears groups.

Morphological Characteristics	Wild Group	Northern Group	Southern and central Group
Seed weight (g)	0.230±0.074 a	0.086±0.007 b	0.106±0.012 b
Seed thickness (cm)	0.640±0.066 a	0.476±0.021 c	0.537±0.022 b
Seed length (cm)	0.966±0.080 a	0.828±0.038 b	0.877±0.031 b
Seed width (cm)	0.744±0.092 a	0.549±0.027 c	0.630±0.029 b
Length-width ratio of seed	1.303±0.053 b	1.515±0.022 a	1.398±0.093 ab
Weight of seed kernel (g)	0.074±0.024 ab	0.056±0.006 b	0.078±0.006 a
Weight of hull and testa (g)	0.156±0.050 a	0.030±0.002 b	0.028±0.008 b
Thickness of seed kernel (cm)	0.369±0.046 ab	0.349±0.015 b	0.401±0.013 a
Length of seed kernel (cm)	0.490±0.048 a	0.506±0.015 a	0.520±0.017 a
Width of seed kernel (cm)	0.550±0.065 a	0.447±0.016 b	0.518±0.017 a
Length-width ratio of seed kernel	0.894±0.019 c	1.135±0.033 a	1.007±0.047 b

Different lowercases indicated significant difference at level of 0.05 by Least-Significant Difference Test (LSD).

### Genetic analysis

In total, 103 reproducible fragments, of which 91 were polymorphic (84.47%), were produced in amplification for 218 samples from 11 Job’s tears accessions by 10 selected ISSR primers (Table 3). The number of polymorphic fragments ranged from 8 (UBC 814) to 13 (UBC 874), with an average of 10.3 for each primer.

**Table 3:** Primers used for genetic analysis of Job’s tears accessions.

Primer	Sequence	T_A_ (°C)	N_PF_/N_F_	PPF (%)
UBC814	(CT)_8_A	54	8/8	100
UBC824	(TC)_8_G	54	10/10	100
UBC825	(AC)_8_T	54	8/11	72.7
UBC826	(AC)_8_C	54	7/10	70.0
UBC827	(AC)_8_G	52	7/10	70.0
UBC842	(GA)_8_YG	52	9/10	90.0
UBC857	(AC)_8_YG	55	7/9	77.8
UBC874	(CCCT)_4_	50	12/13	92.3
UBC886	VDV(CT)_7_	54	8/10	80.0
UBC891	HVH(TG)_7_	54	11/12	91.7

Y = (C, T); V = (A, C, G); D = (A, G, T); H = (A, C, T); T_A_: Annealing temperature; N_PF_: number of polymorphic fragments; N_F_: number of amplified fragments; PPF: percentage of polymorphic fragments.

The Neiʼs expected heterozygosity (*h*), Shannon's Information index (*I*) and percentage of polymorphic fragments (PPF) of all accessions were 0.2051, 0.3261 and 84.47%, respectively. The genetic diversity was different among all accessions. As seen in Table 4, lower genetic diversity (*h* = 0.0335, *I* = 0.0492, PPF = 8.74%) was detected in wild accessions compared to the cultivated accessions (*h* = 0.0753, *I* = 0.1128, PPF = 22.22%). The wild accession ZJLD showed the lowest genetic diversity (*h* = 0.0254, *I* = 0.0337, PPF = 6.80%), while the cultivated accession ZJTS showed the highest genetic diversity (*h* = 0.1325, *I* = 0.1943, PPF = 34.95%). The genetic diversity level of ZJJY (*h* = 0.0330, *I* = 0.0502, PPF = 11.65%) was the lowest among the cultivated accessions, and almost equal to the genetic diversity (*h* = 0.0335, *I* = 0.0492, PPF = 8.74%) of wild accessions.

**Table 4:** Genetic diversity parameters of Job’s tears accessions.

Accession	*h*	*I*	PPF (%)
Wild accession			
SCCD	0.0415	0.0606	10.68
ZJLD	0.0254	0.0377	6.80
Mean	0.0335±0.0114	0.0492±0.0162	8.74±2.74
Cultivated accession			
LNYX	0.0927	0.1398	28.16
SDHZ	0.1112	0.1638	30.10
AHBZ	0.0736	0.1104	21.36
ZJJY	0.0330	0.0502	11.65
FJPC	0.0924	0.1386	27.18
ZJTS	0.1325	0.1943	34.95
GZXR	0.0540	0.0822	16.50
YNSZ	0.0392	0.0603	14.56
GXXL	0.0489	0.0755	15.53
Mean	0.0753±0.0343	0.1128±0.0496	22.22±8.16
All accession			
Mean	0.0677±0.0352	0.1012±0.0515	20.36±9.43

*h*: Neiʼs expected heterozygosity

*I*: Shannon's Information index

PPF: percentage of polymorphic fragments.

**Fig 4:**
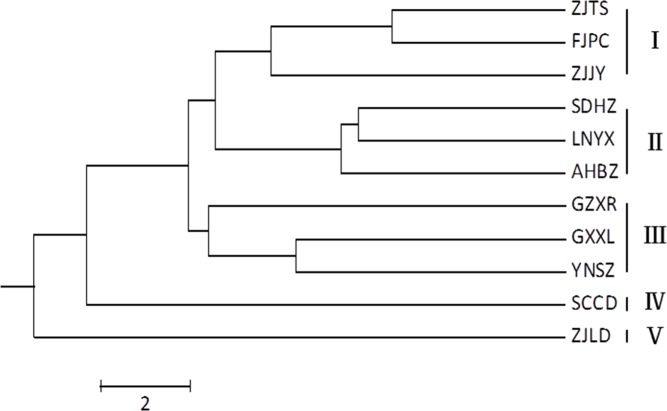
UPGMA dendrogram illustrating the genetic relationships among Job’s tears accessions constructed by ISSR data.

In the UPGMA dendrogram (Fig 4), the Job’s tears accessions were clustered into five clades. The wild accessions of SCCD and ZJLD, which were genetically distant from the cultivated accessions, formed clade IV and clade V, respectively. The genetic relationships between cultivated accessions were closer than those of wild accessions. The cultivated accessions were clustered into three clades. Clade I consisted of three cultivated accessions of ZJTS, FJPC, and ZJJY from central China, and clade II consisted of three cultivated accessions of SDHZ, LNYX, and AHBZ from northern China. Three cultivated accessions of GZXR, GXXL, and YNSZ from southern China formed clade III. Although the central accessions and southern accessions formed two clades in the ISSR dendrogram, the Mantel test showed high consistency between morphological and genetic variations, with an extremely significant positive correlation coefficient (r = 0.696**).

A strong genetic differentiation (G_ST_ = 0.6702) was found among all accessions. The analysis of molecular variance (AMOVA) also showed that 73.42% of total genetic variability occurred among all accessions, indicating significant genetic differentiation of Job’s tears. Most of the genetic diversity of clade III (G_ST_ = 0.6447) and clades IV and V (G_ST_ = 0.7442) was detected among accessions. Relatively low G_ST_ values of 0.4313 and 0.3770 in clade I and clade II, respectively, indicated that most genetic diversity was detected within accessions. Compared with the low gene flow (N_m_) of 0.2460 in all accessions, the gene flow of subdivided cultivated clades was relatively higher, ranging from 0.2756 in clade III to 0.8262 in clade II; the gene flow of clade IV and V (0.1718) was even lower (Table 5).

**Table 5:** Genetic diversity parameters of all Job’s tears accessions and subdivided clades.

Genetic diversity parameters	All accessions	Clade I	Clade II	Clade III	Clades IV and V
Total gene diversity: H_t_	0.2052	0.1512	0.1485	0.1333	0.1309
Gene diversity within accessions: H_S_	0.0677	0.0860	0.0925	0.0474	0.0335
Coefficient of gene differentiation: G_ST_	0.6702	0.4313	0.3770	0.6447	0.7442
Gene flow: N_m_	0.2460	0.6591	0.8262	0.2756	0.1718
Neiʼs expected heterozygosity: *h*	0.2051	0.1512	0.1480	0.1333	0.1309
Shannon's Information index: *I*	0.3261	0.2318	0.2298	0.2029	0.1846
Number of polymorphic fragments: PF	87	52	55	44	29
Percentage of polymorphic fragments: PPF (%)	84.47	50.49	53.40	42.72	28.16

The Mantel test showed no significant correlation between genetic and geographic distance matrices (r = 0.184, P = 0.143) in all accessions, but significant correlation between genetic and geographic distance matrices (r = 0.419, P < 0.01) was detected in the cultivated accessions when the wild accessions were excluded.

### Fatty acid composition

GC-MS analysis detected four fatty acids in Job’s tears seed oil from different accessions. They showed a general order of abundance of oleic acid > linoleic acid > palmitic acid > stearic acid (Table 6), with the one exception being that the linoleic acid content of the ZJJY and ZJTS accessions was slightly higher than the oleic acid content. The unsaturated fatty acid (linoleic acid and oleic acid) content of different accessions was quite high, ranging from 74.08% to 82.28% of the total fatty acids.

**Table 6:** Fatty acid composition of seed oil from different Job’s tears accessions and divided groups.

Accession	Palmitic acid (%)	Linoleic acid (%)	Oleic acid (%)	Stearic acid (%)	Palmitic acid + Linoleic acid/Oleic acid
Wild Group					
SCCD	15.16	31.64	48.65	2.31	0.96
ZJLD	13.62	28.82	53.46	3.03	0.79
Mean	**14.39±1.09 c**	**30.23±2.00 b**	**51.06±3.41 a**	**2.67±0.51 ab**	**0.88±0.12 c**
Northern Group					
LNYX	16.66	37.20	42.26	2.49	1.27
SDHZ	16.96	34.76	44.04	2.43	1.17
AHBZ	16.49	34.48	45.97	2.02	1.11
Mean	**16.70±0.24 b**	**35.48±1.50 a**	**44.09±1.86 b**	**2.31±0.26 b**	**1.19±0.08 b**
Central Group					
ZJJY	17.77	39.52	39.15	2.47	1.46
FJPC	18.12	35.33	38.75	2.81	1.38
ZJTS	18.15	40.50	37.07	2.50	1.58
Mean	**18.01±0.21 a**	**38.45±2.75 a**	**38.32±1.10 c**	**2.59±0.19 b**	**1.47±0.10 a**
Southern Group					
GZXR	18.60	36.18	40.17	3.61	1.36
YNSZ	18.14	36.44	41.49	2.98	1.32
GXXL	17.35	34.85	43.73	3.11	1.19
Mean	**18.03±0.63 a**	**35.82±0.85 a**	**41.80±1.80 bc**	**3.24±0.33 a**	**1.29±0.09 ab**

Different lowercases indicated significant difference at level of 0.05 by Least-Significant Difference Test (LSD). Mean values and significant differences of fatty acid composition of divided groups were given in boldface. For divided groups see Fig 3.

**Fig 5:**
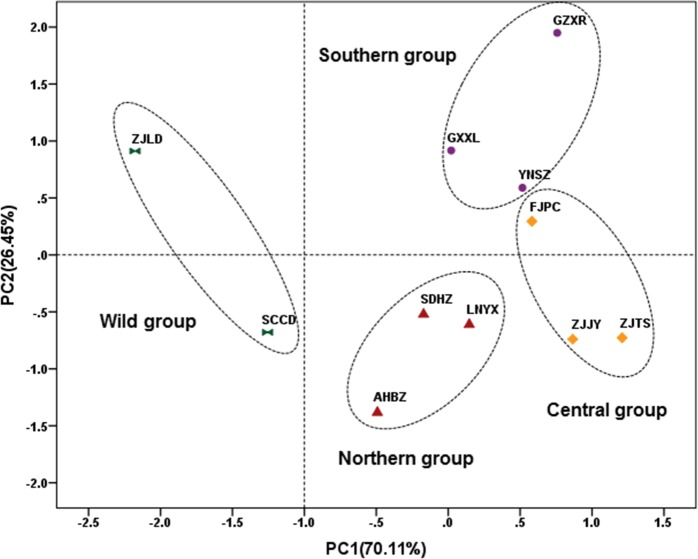
PCA scatter plot of Job’s tears accessions constructed by fatty acid composition of seed oil.

In the PCA scatter plot constructed by the fatty acid compositions of seed oil, the Job’s tears accessions can be divided into four groups: the wild group (SCCD and ZJLD), the northern group (LNYX, SDHZ, and AHBZ), the central group (ZJJY, FJPC, and ZJTS), and the southern group (GZXR, YNSZ, and GXXL) (Fig 5). The grouping pattern is identical with that of ISSR analysis, and showed an extremely significant positive correlation (r = 0.675**) with the genetic variation. The wild group was mainly characterized by low palmitic acid (14.39%) and linoleic acid (30.23%) and high oleic acid (51.06%). The palmitic acid content of the northern group (16.70%) was significantly lower than that of the central group (18.01%) and southern group (18.03%). The southern group differed from the central and northern groups in its high stearic acid content (3.24%) (Table 6). The proportions of palmitic acid and linoleic acid to oleic acid were recorded as 1.47 in the central group, 1.29 in the southern group, 1.19 in the northern group, and 0.88 in the wild group.

### Triglyceride composition

HPLC-ELSD analysis showed that the content of nine triglycerides in seed oil were significantly different in the 11 Job’s tears accessions (Table 7). 1,2-Linolein-3-olein, 1,2-olein-3-linolein, 1-palmitin-2-olein-3-linolein, and triolein were the main components of the seed oil. Their content varied from 17.69% (SCCD) to 27.13% (ZJJY), 22.28% (SDHZ) to 29.29% (ZJLD), 8.04% (ZJLD) to 15.40% (GZXR and GXXL), and 13.56% (GXXL) to 30.79% (SCCD), respectively. The other five triglycerides only represented 13.66% to 23.86% of the total triglycerides.

**Table 7:** Triglyceride composition of seed oil from different Job’s tears accessions and divided groups.

Accession	1 (%)	2 (%)	3 (%)	4 (%)	5 (%)	6 (%)	7 (%)	8 (%)	9 (%)
Wild Group									
SCCD	1.61±0.02 f	17.69±0.10 h	3.19±0.01 g	25.97±0.10 b	9.86±0.07 f	1.00±0.09 f	30.79±0.08 a	9.16±0.05 b	0.74±0.11 d
ZJLD	2.18±0.02 e	18.51±0.26 g	2.66±0.07 h	29.29±0.09 a	8.04±0.09 g	0.75±0.23 g	30.50±0.25 a	7.29±0.07 de	0.78±0.20 cd
Mean	**1.90±0.40 c**	**18.10±0.58 c**	**2.92±0.37 b**	**27.63±2.34 a**	**8.95±1.29 c**	**0.87±0.17 c**	**30.65±0.20 a**	**8.23±1.32 b**	**0.76±0.03 c**
Northern Group									
LNYX	4.57±0.05 b	21.21±0.10 f	6.04±0.14 e	23.55±0.14 c	12.95±0.06 d	1.57±0.04 de	19.30±0.08 b	9.49±0.26 b	1.30±0.09 ab
SDHZ	5.02±0.10 a	21.95±0.27 e	6.50±0.13 cd	22.28±0.06 f	13.54±0.08 c	1.71±0.11 cd	18.36±0.09 c	9.43±0.05 b	1.20±0.13 a-d
AHBZ	4.60±0.08 b	21.77±0.14 ef	4.54±0.03 f	23.45±0.13 c	12.14±0.22 e	1.41±0.04 e	19.66±0.47 b	10.96±0.35 a	1.47±0.23 a
Mean	**4.73±0.25 a**	**21.64±0.39 b**	**5.69±1.03 a**	**23.10±0.71 b**	**12.88±0.70 b**	**1.57±0.15 b**	**19.11±0.67 b**	**9.96±0.86 a**	**1.32±0.14 a**
Central Group									
ZJJY	4.61±0.08 b	27.13±0.53 a	6.70±0.33 bc	22.90±0.49 de	14.48±0.48 b	2.16±0.08 a	14.29±0.10 e-g	6.80±0.33 e	0.93±0.26 b-d
FJPC	4.27±0.08 c	25.74±0.38 cd	6.48±0.05 cd	23.43±0.30 cd	13.47±0.23 cd	1.68±0.04 cd	16.12±0.25 d	7.86±0.31 cd	0.95±0.35 a-d
ZJTS	4.92±0.03 a	26.70±0.28 ab	7.04±0.21 a	22.73±0.18 ef	14.12±0.08 b	2.00±0.08 ab	14.83±0.09 e	6.75±0.04 e	0.92±0.53 b-d
Mean	**4.60±0.33 ab**	**26.52±0.71 a**	**6.74±0.28 a**	**23.02±0.37 b**	**14.02±0.51 b**	**1.95±0.25 a**	**15.08±0.94 c**	**7.14±0.63 b**	**0.93±0.02 b**
Southern Group									
GZXR	4.01±0.12 d	26.04±0.54 bc	6.69±0.13 bc	23.83±0.08 c	15.40±0.19 a	1.74±0.20 cd	14.09±0.75 fg	6.89±0.60 e	1.31±0.44 ab
YNSZ	4.27±0.06 c	25.37±0.19 cd	6.28±0.23 de	23.67±0.51 c	15.34±0.43 a	2.06±0.26 ab	14.40±0.73 ef	7.41±0.20 de	1.21±0.14 a-d
GXXL	3.95±0.17 d	25.31±0.90 d	6.83±0.31 ab	23.52±0.64 c	15.40±0.54 a	1.92±0.05 bc	13.56±0.74 g	8.29±1.39 c	1.23±0.32 a-c
Mean	**4.08±0.17 b**	**25.57±0.41 a**	**6.60±0.28 a**	**23.67±0.16 b**	**15.38±0.03 a**	**1.91±0.16 ab**	**14.02±0.42 c**	**7.53±0.71 b**	**1.25±0.05 a**

Different lowercases indicated significant difference at level of 0.05 by Least-Significant Difference Test (LSD). Mean values and significant differences of triglyceride composition of divided groups were given in boldface. For divided groups see Fig 4. 1: trilinolein; 2: 1,2-linolein-3-olein; 3: 1,2-linolein-3-palmitin; 4: 1,2-olein-3-linolein; 5: 1-palmitin-2-olein-3-linolein; 6: 1,3-palmitin-2-linolein; 7: triolein; 8: 1,2-olein-3-palmitin; 9: 1-palmitin-2-linolein-3-stearin.

**Fig 6:**
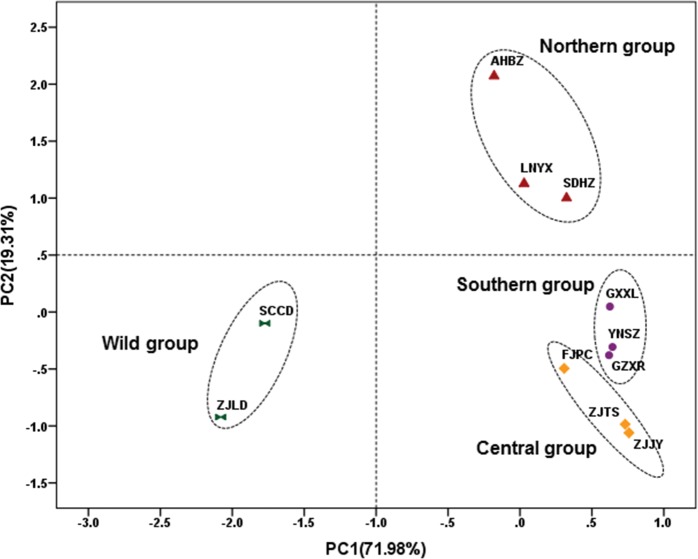
PCA scatter plot of Job’s tears accessions constructed by triglyceride composition of seed oil.

According to the PCA scatter plot constructed by the triglyceride compositions of seed oil, the Job’s tears accessions can be divided into four groups: the wild group (SCCD and ZJLD), the northern group (LNYX, SDHZ, and AHBZ), the central group (ZJJY, FJPC, and ZJTS), and the southern group (GZXR, YNSZ, and GXXL) (Fig 6). The grouping pattern is also identical with the one clustered by ISSR analysis. Furthermore, an extremely significant positive correlation was found between ISSR and HPLC-ELSD data (r = 0.805**). Compared with the other three groups, the wild group was significantly richer in 1,2-olein -3-linolein (27.63%) and triolein (30.65%), and poor in the other six triglycerides, except 1,2-olein-3-palmitin. The northern group can be separated from the southern and central groups based on its higher triolein (19.11%) and 1,2-olein-3-palmitin (9.96%) content, and its lower 1,2-linolein-3-olein (21.64%) content. The southern group possessed higher 1-palmitin-2-olein-3-linolein (15.38%) and 1-palmitin-2-linolein-3-stearin (1.25%) content than the central group (Table 7).

### Inhibition rates of Job’s tears oil for T24 cells

**Fig 7:**
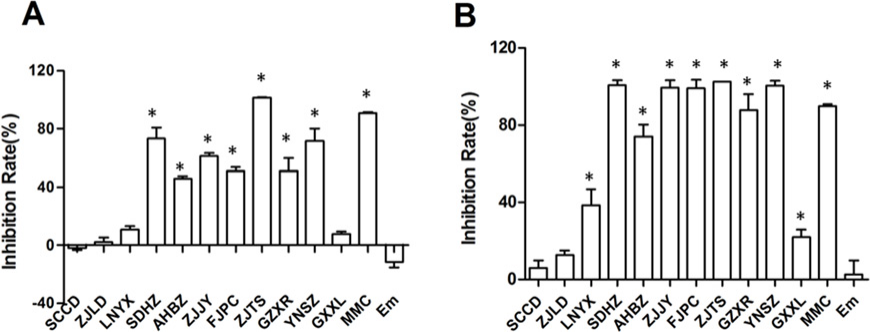
Inhibition rates of Job’s tears seed oil for T24 cells. (A: oil concentration = 0.33%; B: oil concentration = 0.5%; Em: emulsifier group; *: significant difference from control group)

The inhibition rates of Job’s tears seed oil were significantly different among accessions, varying from -1.80% (SCCD) to 101.59% (ZJTS) at a concentration of 0.33% and from 5.84% (SCCD) to 102.42% (ZJTS) at a concentration of 0.5% (Fig 7). There was no significant difference between the emulsifier group and the control group at both concentrations (Fig 7).

Compared with the control group, the seed oil of almost all cultivated accessions significantly inhibited the proliferation of T24 cells in a concentration-dependent manner, while the seed oil of wild accessions (SCCD and ZJLD) showed no repressive effect at either concentration. The oil of the ZJTS accession showed the most potent repressive effect against T24 cells, with an inhibition rate of 101.59% at a concentration of 0.33% (Table 8). The GXXL, LNYX, and AHBZ accessions showed significantly lower inhibition rates for T24 cells than the other cultivated accessions. According to the groups divided by genetic variation or chemical compositions, the inhibition rates of Job’s tears seed oil were ordered as central group > southern group ≈ northern group > wild group. However, the differences among the central, southern, and northern groups were not significant (Table 8).

**Table 8:** Inhibition rates of Job’s tears seed oil for T24 cells (Mean ± SD, %).

Accession	0.33%	0.5%
Wild Group		
SCCD	-1.80±2.37 fg	5.84±6.84 f
ZJLD	2.38±4.92 ef	12.69±3.90 ef
Mean	**0.29±2.95 b**	**9.27±4.84 b**
Northern Group		
LNYX	10.96±3.89 e	38.50±14.42 d*
SDHZ	73.18±13.59 b*	100.66±4.71 ab*
AHBZ	45.75±2.98 d*	74.06±10.56 c*
Mean	**43.30±31.18 ab**	**71.07±31.18 a**
Central Group		
ZJJY	61.43±2.94 bc*	99.32±6.62 ab*
FJPC	50.85±5.02 cd*	99.14±7.50 ab*
ZJTS	101.59±0.44 a*	102.42±0.24 a*
Mean	**71.29±26.77 a**	**100.29±1.85 a**
Southern Group		
GZXR	50.96±15.61 cd*	87.69±14.31 bc*
YNSZ	71.85±14.63 b*	100.48±4.39 ab*
GXXL	7.69±3.23 ef	22.08±6.62 e*
Mean	**43.50±32.72 ab**	**70.09±42.07 a**
Emulsifier	-11.43±6.44 g	2.46±12.63 f
MMC(100μg/mL)	90.85±1.08 a*	89.84±1.63 ab*

Different lowercases indicated significant difference at level of 0.05 by Least-Significant Difference Test (LSD)

*: significant difference from control group.

Mean values and significant differences of inhibition rates of divided groups were given in boldface. For divided groups see Figs 3–5.

A significant correlation can be seen between inhibition rates and content of single fatty acids. The inhibition rates showed a significant or extremely significant positive correlation with palmitic acid content (r = 0.777**, 0.691*; concentration = 0.5%, 0.33%) and linoleic acid content (r = 0.693*, 0.706*; concentration = 0.5%, 0.33%), and a significant or extremely significant negative correlation with oleic acid content (r = -0.765**, -0.709*, concentration = 0.5%, 0.33%) (Table 9). The inhibition rates displayed an extremely significant positive correlation with the proportion of palmitic acid and linoleic acid to oleic acid (r = 0.765** at a concentration of 0.5% and r = 0.745** at a concentration of 0.33%).

**Table 9:** Correlation coefficients between inhibition rates and fatty acid content.

	palmitic acid	linoleic acid	oleic acid	stearic acid
Inhibition rate 1	0.777**	0.693*	-0.765**	-0.014
Inhibition rate 2	0.691*	0.706*	-0.709*	-0.084

*: Significant difference (P < 0.05);

**: Significant difference (P < 0.01).

Inhibition rate 1: Inhibition rate at a concentration of 0.5%; Inhibition rate 2: Inhibition rate at a concentration of 0.33%.

The inhibition rates at both concentrations demonstrated a significant positive correlation with 1,2-linolein-3-olein, 1,2-linolein-3-palmitin and 1,3-palmitin-2-linolein content, and a significant negative correlation with 1,2-olein-3-linolein and triolein content (Table 10). The inhibition rates at a concentration of 0.5% showed an extremely significant positive correlation with trilinolein content (r = 0.782**) and a significant positive correlation with 1-palmitin-2-olein-3-linolein content (r = 0.640*). Significant positive correlation (r = 0.726*) was detected between trilinolein content and the inhibition rates at a concentration of 0.33%.

**Table 10:** Correlation coefficients between inhibition rates and triglyceride content.

	1	2	3	4	5	6	7	8	9
Inhibition rate 1	0.782**	0.734*	0.695*	-0.706*	0.640*	0.719*	-0.716*	-0.276	0.266
Inhibition rate 2	0.726*	0.677*	0.638*	-0.649*	0.567	0.686*	-0.635*	-0.325	0.149

*: Significant difference (P < 0.05)

**: Significant difference (P < 0.01).

Inhibition rate 1: Inhibition rate at a concentration of 0.5%; Inhibition rate 2: Inhibition rate at a concentration of 0.33%. 1: trilinolein; 2: 1,2-linolein-3-olein; 3: 1,2-linolein-3-palmitin; 4: 1,2-olein-3-linolein; 5: 1-palmitin-2-olein-3-linolein; 6: 1,3-palmitin-2-linolein; 7: triolein; 8: 1,2-olein-3-palmitin; 9: 1-palmitin-2-linolein-3-stearin.

## Discussion

The seed oil of cultivated Job’s tears accessions significantly inhibited the proliferation of T24 cells. Triglycerides were confirmed as the main active components of Job’s tears seed oil [31]. In this study, the anti-proliferative effects of Job’s tears seed oil on T24 cells were shown to be significantly different among accessions. The inhibition rates showed a significantly positive correlation with single triglyceride components rich in palmitic and linoleic acid, and a significantly negative correlation with single triglyceride components rich in oleic acid. The correlation between inhibition rates and free fatty acid content also indicated the same results, i.e., the inhibition rates showed a significantly positive correlation with palmitic and linoleic acid content and a significantly negative correlation with oleic acid content. Therefore, the higher the proportion of palmitic acid and linoleic acid to oleic acid in Job’s tears seed oil, the better its anti-proliferative effect on T24 cells.

Several studies have indicated that palmitic and linoleic acid can inhibit the growth of cancer cells, and that oleic acid has the opposite effect. Linoleic acid has been verified to suppress the growth of human colorectal cancer cells [49,50], human myeloid leukemia cells [51], and human pancreatic cancer cells [51,52]. In contrast, oleic acid stimulates the proliferation of human myeloid leukemia cells [51], human pancreatic cancer cells [51,52], and human breast cancer cells [53,54]. Palmitic acid can suppress the growth of human breast cancer cells [54,55], human osteosarcoma cells [56], and rat insulinoma cells [57]. Four free fatty acids, that is, palmitic, stearic, oleic, and linoleic acids, were previously found to be the antitumor components of Job’s tears seed oil [58]. The current study demonstrated that the overall fatty acid composition of Job’s tears seed oil was more important than the content of single fatty acid for inhibiting the proliferation of cancer cells. The accession ZJTS, with a proportion of palmitic acid and linoleic acid to oleic acid greater than 1.5, showed the most potent anti-proliferative effect. Wild accessions with a proportion lower than 1 had no repressive effect on T24 cells. Therefore, the proportion of palmitic acid and linoleic acid to oleic acid is an important indicator of quality in Job’s tears.

The fatty acid and triglyceride compositions were significantly different among the 11 Job’s tears accessions, and the chemical profiles showed an extremely significant positive correlation with genetic variation. Typically, the yield and variability of secondary metabolites can be influenced by physiological variations, environmental conditions, geographic variation, genetic factors, and evolution [59]. However, as primary metabolites, the fatty acid and triglyceride compositions were mainly attributed to genetic factors [60–62]. We suggest that the fatty acid and triglyceride compositions of Job’s tears seeds may mainly depend on their genetic background. Genetic improvement may be therefore a feasible action for improving the quality and antitumor efficacy of Job’s tears seeds.

In this study, the 11 Job’s tears accessions were divided into two to three cultivated groups corresponding with geographical locations, and one to two evidently divergent wild groups by seed morphological characteristics, ISSR data, or chemical profiles. The inhibition rates for T24 cells were also different among the divided groups. ISSR markers are stable in all tissues throughout the lives of the plants, and are independent of environmental, pleiotropic, and epistatic effects [63]. The ISSR profiles of Job’s tears demonstrated an extremely significant positive correlation (r = 0.696**) with seed morphological characteristics, indicating that genetic variability underlies the morphological characteristics of Job’s tears seeds. It is generally believed that the morphological and physiological traits of plants can be influenced by environmental conditions and cultural practices [64]. Environmental and cultural factors have little effect on the morphological characteristics of Job’s tears seeds, although the two genetically divergent groups of southern and central accessions were grouped together based on seed morphological characteristics. The seed morphological characteristics are valuable in preliminary selection as they are easy to analyze. In general, yellow-white and soft seeds with moderate weight and size may be best for medicinal purposes.

The genetic diversity of Job’s tears at the species level (*h* = 0.21, *I* = 0.33) and accession level (*h* = 0.07, *I* = 0.10) was lower than that of the Gramineae species *Miscanthus sinensis* (species *h* = 0.30, *I* = 0.46; population *h* = 0.14, *I* = 0.22) and *Elymus sibiricus* (species *h* = 0.27, *I* = 0.41; population *h* = 0.18, *I* = 0.27), as detected by ISSR markers [65,66]. Typically, species with wide distribution and an outcrossing mating system possess higher genetic diversity than those with restricted distribution and selfing or mixed mating systems [67–70]. The staminate flowers and pistillate flowers of the same inflorescence of a Job’s tears blossom at different times [71], but pollination can easily occur between inflorescences of the same plant. The natural outcrossing rate of Job’s tears is only about 36% [72]. Therefore, Job’s tears is a plant with a mixed mating system. Furthermore, the accessions used in this study are restricted to their area of occupancy. Thus, the low genetic diversity of Job’s tears may be attributed to its mating system and restricted distribution.

Job’s tears is native to India, Burma, China, and Malaysia [3]. Job’s tears is widely distributed in almost every province throughout China [5]. In China, habitat fragmentation caused by plowing rangeland for agricultural and transportation purposes has led to a decrease in the Job’s tears population [73]. Species with small population sizes and habitat fragmentation commonly demonstrate low genetic variation [74–76]. Therefore, the extremely low genetic diversity of wild accessions could be due to habitat fragmentation and small accession numbers. Additionally, the low germination rate of wild accessions is another factor that caused its low genetic diversity. As observed in this study, the seeds of wild accessions did not germinate in the laboratory unless they were dehulled, while the seeds of cultivated accessions reached almost a 100% germination rate.

The low genetic diversity of cultivated Job’s tears accessions can be attributed to its long-term domestication. Domestication has led to an overall reduction in the genetic diversity of many crops by replacing landraces and traditional varieties with less diverse modern cultivars and hybrids (although this increases their productivity dramatically) [77]. The southern region is the primary origin center of Job’s tears in China, and the central and northern regions are considered secondary centers [78]. The cultivation history of Job’s tears in southern China is longer than that in central and northern China [79]. The lower genetic diversity of southern accessions can be attributed to the stronger artificial selection they have experienced.

The genetic structure of a species is affected by multiple evolutionary factors including the breeding system, gene flow (pollen and seed dispersal), life form, and geographic range [80]. Job’s tears is an annual plant with a low outcrossing rate, which tends to have a high G_ST_ value. Typically, N_m_ > 1 is considered to be sufficient to prevent accession differentiation [81]. The low gene flow (N_m_ = 0.2460) of all Job’s tears accessions is not sufficient to counteract the genetic differentiation caused by genetic drift, and has resulted in great genetic differentiation (G_ST_ = 0.6702) among all accessions. The Job’s tears resources of China can be divided into three ecotypes (northern China, central China, and southern China) according to morphological, biological, and agronomic traits [78], which supports the geographic grouping pattern of Job’s tears accessions in this study. The high genetic differentiation among southern accessions of clade III (G_ST_ = 0.6447) can be attributed to low gene flow (N_m_ = 0.2756), while the low genetic differentiation among northern accessions of clade I (G_ST_ = 0.4313) and central accessions of clade II (G_ST_ = 0.3770) can be attributed to relatively higher gene flow (N_m_ = 0.6591, 0.8262). The geographic pattern of cultivated accessions indicated that gene flow was restricted by geographic distance. Job’s tears is an anemophilous plant, but the distance between southern, central, and northern accession regions is too great for the pollen to cover. In addition, the seeds are too heavy to be carried to distant regions by wind, and they fall only around their mother plants, which makes seed dispersal even more difficult than pollen dispersal without artificial spread. The genetic structure of Job’s tears is affected by its breeding system, gene flow, life form, and geographic range.

The low genetic diversity of Job’s tears accessions was identified in this study and supported by genetic diversity estimates for Chinese and Korean accessions [28–30]. Therefore, it might be relatively difficult to obtain higher quality Job’s tears through breeding with present accessions. The central accessions ZJTS, ZJJY, and FJPC, which were characterized by yellow-white and soft seeds with moderate weight and size, may be a better choice for medicinal purposes. Further work should emphasize the importance of breeding and selecting for Job’s tears that contain higher proportions of palmitic acid and linoleic acid to oleic acid in their seed oil. Meanwhile, more wild and cultivated accessions should be collected as breeding materials and improved by mutation breeding or genetic engineering to increase the genetic diversity of Job’s tears.

## Supporting Information

S1 Table Seed morphological characteristics of Job’s tears accessions. (XLSX)
